# Distinct Influences of Social Activity and Social Connectedness on Depressive Symptoms in Older Adult Couples

**DOI:** 10.1093/geroni/igab046.1220

**Published:** 2021-12-17

**Authors:** Joel Steele, Lyndsey Miller, Chao-Yi Wu, Hiroko Dodge, Jeffrey Kaye, Karen Lyons

**Affiliations:** 1 Portland State University, Portland, Oregon, United States; 2 Oregon Health & Science University, Portland, Oregon, United States; 3 Boston College, Chestnut Hill, Massachusetts, United States

## Abstract

This study aimed to simultaneously examine the associations between social activity and connectedness and depressive symptoms in older adult couples. Using SEM and data from 116 community-dwelling couples (age 76.18 ± 8.49), we found that engagement in social activities was associated with lower depressive symptoms in men (p = 0.014), whereas more close friendships were associated with lower depressive symptoms in women (p = 0.018), controlling for partner effects, age, education, and cognitive function (CFI: 1.00, TLI: 1.35, RMSEA: 0.00 [0.00, 0.08]). Unexpectedly, we also found better female physical health to be associated with greater depressive symptoms in males (p = 0.029). When examined as dyadic physical health, more incongruence between the physical health of partners was associated with greater depressive symptoms in men (p = 0.007). Discussion will focus on distinct influences of social activity and connectedness on mental health, and the context of gender, marriage, and dyadic health.

